# Ageing and the immune system in vivo: commentary on the 16th session of British Society for Immunology Annual Congress, Harrogate, December 2004

**DOI:** 10.1186/1742-4933-2-5

**Published:** 2005-02-22

**Authors:** Richard Aspinall

**Affiliations:** 1 Dept of Immunology, Imperial College London, Chelsea & Westminster Hospital London, 369 Fulham Road, London. SW10 9NH, UK

## Abstract

The problems associated with the ageing immune system coupled with possible solutions were discussed recently at the British Society for Immunology Annual Congress in Harrogate in December 2004. The session "Ageing and the Immune System in vivo" dealt in details with the immune risk phenotype and the potential methods of reversing the problems of an ageing immune system. This is a commentary on that session.

From an early age our immune system is introduced to a variety of infectious agents through contact with infected individuals. We also deliberately present our immune responses either to attenuated organisms or to components of infectious pathogens in order to provoke a response. A successful response to these organisms when first encountered provides us with immunological memory to enable our immune systems to make more rapidly responses to the same potentially infectious agents at a later date. In theory the longer we live the better our memory responses and our ability to cope with any potential pathogen seen previously. Since there are no completely sterile environments, individuals who have survived to reach a ripe old age must have combated many possible infectious agents, and have an immune system with a prodigious memory component. Only part of this is true. As we age the memory component of our immune system, as measured by the number of memory cells, does increase. Unfortunately this is not accompanied by improved immunity even to infectious agents that have been overcome earlier in life [1-4].

The problems associated with the ageing immune system coupled with possible solutions were discussed recently at the British Society for Immunology Annual Congress in Harrogate in December 2004. The session "Ageing and the Immune System in vivo" dealt in details with the immune risk phenotype and the potential methods of reversing the problems of an ageing immune system.

The concept of the immune risk phenotype was described by Dr Anders Wikby (Jönköping University) who carried out a longitudinal immunological study in order to establish predictive factors for longevity. Dr Anders first worked on a group of normal octogenarians in the OCTA study in Sweden which later became the NONA study as these individuals entered their nineties. Within this group he was able to describe the immune risk phenotype which he characterised by a CD4:CD8 ratio of <1, poor in vitro T cell proliferation, an increase in the number of CD8^+^CD28^- ^cells (or CD8^+^CD28^-^CD27^- ^in the very old), low numbers of B cells and the presence of CD8 T cells which were cytomegalovirus (CMV) tetramer positive. Within both the OCTO and NONA groups Dr Anders was able to show that the immune risk phenotype had some predictive input towards morbidity. This was even more apparent when cognitive impairment was included in the calculations.

The effect of CMV infection on the immune system in the elderly was also discussed by Professor Paul Moss (University of Birmingham) with particular emphasis on the clonal expansion of T cells. Clonally expanded T cells are usually CD8^+ ^and show an increased incidence with age, so far it seems that clonal expansion is not due to malignant transformation but may follow antigen stimulation. The control of CMV within the body is mainly attributed to CD8^+ ^T cells and Professor Moss revealed that in older individuals up to 50% of CD8^+ ^T cells may be specific for CMV as judged by tetramer staining. It is possible that this increased response to CMV may lead to the impairment of responses to other viruses in the aged. This theme of CMV infection and ageing was then continued by Professor Graham Pawelec (University of Tuebingen) who introduced the use of the KLRG-1 marker as a marker of senescence. T cells which are KLRG-1^+ ^do not proliferate in vitro when provided with a stimulus which would induced proliferation in T cells which lack this marker. In elderly individuals fewer CMV tetramer positive CD8^+ ^T cells secreted interferon-γ in Elispot assays when compared with similar cells from younger individuals. Furthermore between 96 and 99%of the CD8+ T cells which stained with the CMV tetramer also expressed the KLRG-1 marker. The theme of CMV infection in the elderly was further continued by Professor Arne Akbar (University College London) whose research group has recently been analyzing telomere lengths in different T cell subsets using a fluorescent staining technique. In this method telomeres are stained with a fluorescently labeled probe and the brighter the fluorescent labeling the longer the telomere. Using this technique Professor Akbar reported that CMV specific CD4+ T cells in older individuals had very short telomeres indicating their limited replicative capacity and that more of these CMV specific CD4+ T cells expressed interferon-γ.

This view of a bleak future for displaying the immune risk phenotype was countered by the last two presentations which discussed different approaches to reversing the defects seen in the immune system with age. Both of these methods centered around reversing the atrophy of the thymus seen with age. Dr Jayne Sutherland, currently at the Anthony Nolan Research Institute, detailed her work carried out whilst with Professor Richard Boyd (Monash University) on reversing age associated thymic atrophy by surgical or chemical castration. Studies in mice revealed that reversal of thymic atrophy followed surgical castration and in humans chemical castration produced changes in the TREC levels which indicated improved thymic output. Functional studies showed that this treatment improved immune function in the treated aged subjects. This theme of improved immunity following reversal of thymic atrophy was carried further in work reported by Dr Richard Aspinall (Imperial College, London). He showed how therapeutic intervention with interleukin 7 and derivates could reverse atrophy of the thymus in old animals and also lead to improved immune function compared with age and sex matched controls. In addition he described work done in the Gambia which suggested a link between the development and enlargement of the thymus after birth and the level of interleukin 7 in breast milk.

Two speakers from the posters presentations were also chosen to present their results. K. Wang from Professor Janet Lord's group (Birmingham) presented work on dehydroepiandrosterone, (DHEA). This steroid hormone declines with age and treatment of NK cells with DHEA increases NK cytotoxicity and further analysis shows that DHEA induces PKC-β translocation and upregulates perforin expression in these cells. D. Silva from Donald Palmers laboratory (Royal Veterinary College) presented work on the expression of neuropeptides in epithelial cells from the thymuses of different species.

The meeting revealed that much needs to be done in characterizing the changes in the immune system which are associated with ageing, and members of the audience were asked whether they were interested in receiving more details about the newly formed "Differentiation and Immunosenesence Affinity Group" associated with the BSI.
